# From the Flight Deck to the Trading Desk: Gamblified Investing Behavior in a Commercial Airline Pilot

**DOI:** 10.7759/cureus.66861

**Published:** 2024-08-14

**Authors:** Piercarlo Minoretti

**Affiliations:** 1 Occupational Health, Studio Minoretti, Oggiono, ITA

**Keywords:** occupational health screening, cognitive behavioral therapy, financial risk-taking, gamblified investing, airline pilots

## Abstract

The convergence of investing and gambling has accelerated with the proliferation of *gamblified* investment products characterized by high volatility. This case report examines a 42-year-old male commercial airline pilot who developed maladaptive engagement with high-risk financial instruments during the COVID-19 pandemic, resulting in significant financial losses. The patient's behavior, marked by an inability to adapt to market conditions and attempts to recoup losses through increasingly speculative investments, mirrors patterns observed in problem gambling. Notably, as demonstrated by proficient performance on the Big Three financial literacy assessment, the patient's elevated financial literacy level failed to serve as a protective factor against problematic speculative behavior. This case highlights potential risk factors in aviation professionals, including personality traits like high extraversion and elevated disposable income. Following cognitive behavioral therapy (CBT), the patient successfully transitioned to more conservative investment strategies, with improvements in psychometric scores. However, his posttreatment score on the National Opinion Research Center Diagnostic Screen for Gambling Problems, while improved, still indicated an at-risk status, necessitating ongoing monitoring. This case underscores the need for enhanced awareness, targeted screening protocols, and tailored interventions within occupational health settings, particularly in safety-critical professions like commercial aviation. Future research should focus on developing comprehensive screening instruments for the early identification of problematic financial behaviors, investigating the long-term efficacy of therapeutic modalities like CBT, and examining the prevalence and safety implications of high-risk financial behaviors among aviators.

## Introduction

While investing and gambling share certain characteristics, such as financial risk, uncertainty, and the potential for monetary gains or losses, they are fundamentally distinct activities [1]. Investing is widely recognized as a prudent strategy for long-term wealth accumulation. In contrast, gambling is frequently linked to addiction and financial detriment, particularly in the form of pathological gambling, a disorder of impulse control marked by recurrent and maladaptive gambling behaviors [2]. However, in recent years, the line between investing and gambling has become increasingly blurred with the emergence of *gamblified *investment products characterized by high price volatility [3-5]. These financial instruments include low-priced, thinly traded penny stocks; highly speculative altcoins in the cryptocurrency market; complex and volatile financial instruments such as leveraged exchange-traded funds (ETFs); and binary options [5]. The proliferation of these products has raised concerns about the potential for harm to investors, particularly those who may be vulnerable to addictive behaviors [6,7].

While traditional gambling has been associated with lower socioeconomic status and educational attainment [8], the potential for high-risk financial behavior among airline pilots presents a more nuanced scenario. Accordingly, certain personality traits observed in airline pilots, such as high extraversion scores (particularly evident in facets like assertiveness, activity, and novelty-seeking) when compared to population norms [9], coupled with high disposable incomes, may potentially serve as risk factors for engaging in a *gamblified *investment style. Furthermore, the low neuroticism scores of pilots compared to their non-pilot counterparts [9], reflecting a high ability to handle stress, fear, and anxiety, may further amplify their confidence in high-risk investment products. A case that has been cited in discussions of risky financial behavior among pilots is that of SilkAir Flight MI 185, although it is important to note that the circumstances surrounding this incident remain subject to debate. On December 19, 1997, this Boeing 737-300 aircraft crashed into the Musi River near Palembang, Southern Sumatra, while en route from Jakarta, Indonesia, to Singapore, resulting in the loss of all 104 lives on board [10]. One hypothesis proposed during the investigation suggested that the crash may have been intentionally caused by the pilot-in-command [11]. This theory was partially based on reports that the captain had been involved in stock trading activities and had allegedly incurred significant financial losses before the incident [11].

Here, we present the case of a commercial airline pilot who developed a pattern of maladaptive engagement with high-risk financial instruments, exhibiting characteristics akin to gambling addiction, following the widespread grounding of passenger aircraft during the COVID-19 pandemic. This case underscores the imperative for enhanced awareness and targeted screening of problem gambling among airline pilots, given the safety-critical nature of their occupation.

## Case presentation

In April 2022, a 42-year-old male commercial airline pilot, with over 6,000 hours of flight experience, presented during a routine occupational health evaluation, reporting progressive sleep disturbances. Initially, he experienced sleep onset and maintenance insomnia, characterized by difficulty initiating and sustaining sleep. Over time, these disturbances evolved into a more severe and persistent pattern of chronic insomnia. The progression was marked by an increasing frequency of nocturnal awakenings and a reduction in total sleep time. As the condition worsened, the patient began to experience significant daytime sequelae, including impaired attention. He attributed these issues to his escalating involvement in online investment activities, primarily focused on high-volatility financial instruments such as leveraged ETFs and penny stocks. This pattern of behavior resulted in substantial financial losses exceeding 150,000 euros over the preceding months. The pilot disclosed a longstanding interest in finance and online investing, which intensified significantly during the COVID-19 pandemic when commercial aircraft were grounded, affording him increased discretionary time. During this period of inactivity, he monitored U.S. markets at least five times per hour during Wall Street trading hours. His initial venture into high-risk investing commenced in April 2020 with a leveraged ETF on the volatility index (VIX), which yielded rapid monetary gains during stock market sell-offs. However, the subsequent V-shaped market recovery, coupled with his persistently bearish market outlook, rapidly eroded these gains and resulted in substantial losses. In an attempt to recoup these losses, the patient initiated long positions in penny stocks, reportedly falling victim to *pump-and-dump* schemes, further exacerbating his financial predicament. Despite resuming normal piloting duties post-pandemic, he reported an increasingly compulsive involvement in financial markets, engaging in trading activities almost daily. During this period, he frequently checked market conditions via mobile applications whenever possible, depending on his work responsibilities. He acknowledged that this behavior was potentially detrimental to his well-being.

**Figure 1:**
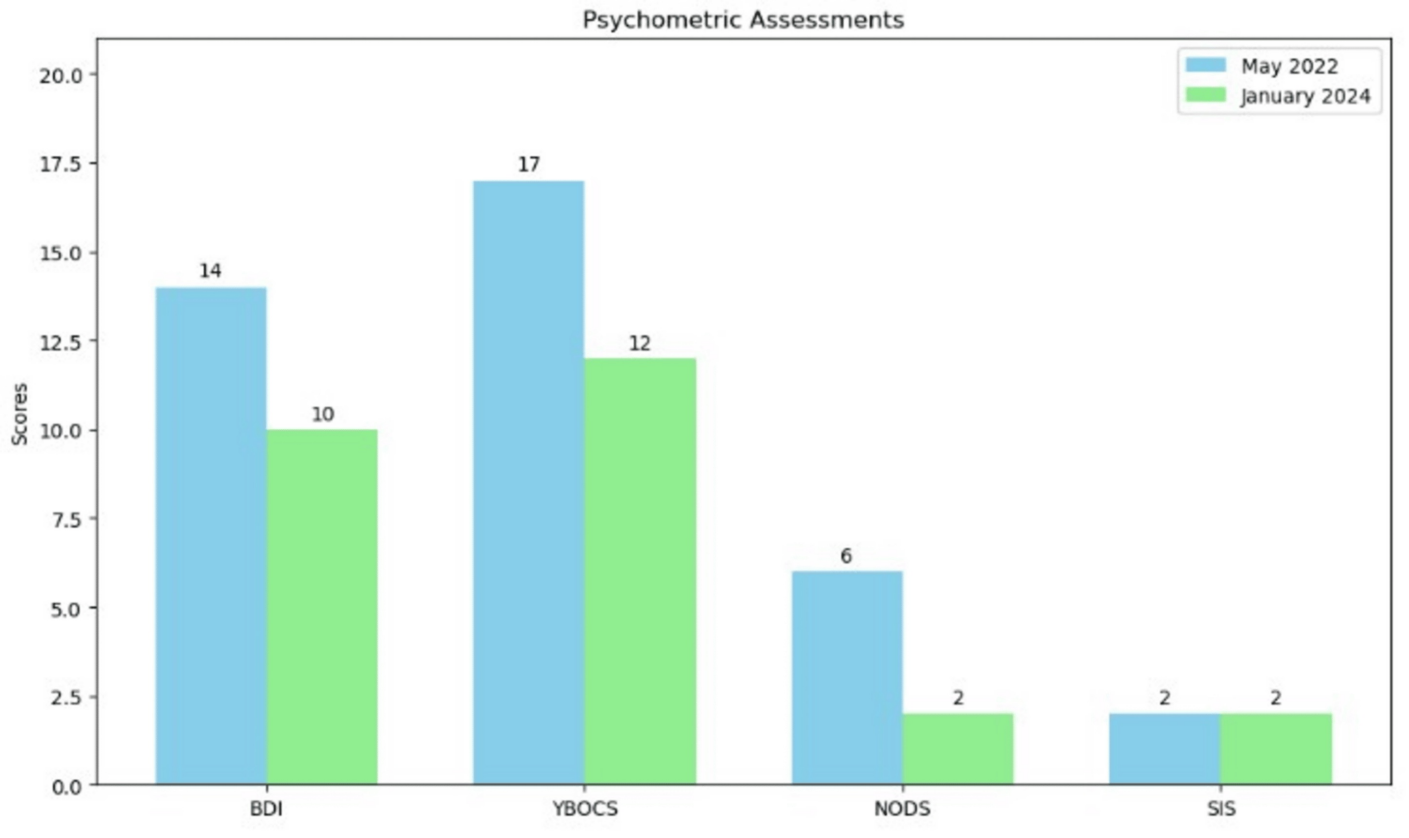
Psychometric assessment scores for the airline pilot at two time points: May 2022 (initial assessment) and January 2024 (follow-up assessment after intervention) The graph shows scores for four different psychometric tests, demonstrating the changes in the pilot's psychological state over time. BDI, Beck Depression Inventory; YBOCS, Yale-Brown Obsessive Compulsive Scale; NODS, National Opinion Research Center Diagnostic Screen for Gambling Problems; SIS, Beck's Suicide Intent Scale

An appointment with a psychologist was scheduled, and at the time of the initial assessment in May 2022, the pilot's psychometric scores were as follows: Beck Depression Inventory (BDI) [12] scored 14, indicating mild depression (range: 0-63); Yale-Brown Obsessive Compulsive Scale (YBOCS) [13] scored 17, indicating moderate symptoms (range: 0-40); National Opinion Research Center Diagnostic Screen for Gambling Problems (NODS) [14] scored 6, positive for pathological gambling (range: 0-10); and the 15-item Beck's Suicide Intent Scale (SIS) [15] scored 2, indicating no suicidal intent (range: 0-30). Notably, he also demonstrated a strong grasp of basic financial concepts, having correctly answered all three of the *Big Three *financial literacy questions [16]. These questions cover three essential concepts: compound interest, inflation, and risk diversification. The compound interest question asks about the growth of money in a savings account over time. The inflation question tests an understanding of how inflation affects purchasing power. Finally, the risk diversification question assesses knowledge of investment risk by comparing single-stock investments to mutual funds [16]. Given the psychometric findings, the patient was advised to receive psychotherapeutic support in the form of online cognitive behavioral therapy (CBT) sessions, which could be accommodated within his professional schedule. The treatment consisted of 15 weekly sessions, each lasting approximately 60 minutes aiming to achieve abstinence from trading activities through a structured approach that includes tracking investing behavior, conducting functional analyses, and developing specific skills to stop gamblified investing. The therapy focused on cognitive restructuring to challenge and modify investment-related thought patterns, while also addressing environmental factors that contributed to the behavior. Early sessions emphasized self-monitoring and reward systems for abstinence, gradually progressing to more complex strategies for managing triggers. The pilot demonstrated a favorable response to the intervention. While maintaining an active interest in finance, he transitioned to more prudent investment strategies, primarily focusing on short-term government and investment-grade corporate bonds. A follow-up assessment conducted in January 2024, following the completion of CBT sessions, yielded the following psychometric results: BDI, 10 (normal; range: 0-63); YBOCS, 12 (mild; range: 0-40); NODS, 2 (at-risk subject with an increased likelihood of progression to problem or pathological gambling); and 15-item SIS, 2 (no suicidal intent). These results indicate a significant improvement in the patient's psychological state (Figure 1), underscoring the efficacy of the implemented therapeutic intervention.

## Discussion

Albeit still subject to debate [11], the tragic case of SilkAir Flight MI 185 serves as a sobering reminder of the potential consequences of uncontrolled financial risk-taking among airline pilots. This case report presents a pilot who developed a pattern of maladaptive engagement with high-risk financial instruments during the COVID-19 pandemic, which persisted and led to significant financial losses over an extended period. Notably, the patient's inability to adapt to changing market conditions and his attempts to recoup losses through increasingly speculative investments mirror the behavior commonly observed in problem gambling. Paradoxically, the patient's elevated level of financial literacy, as demonstrated by his proficient performance on the "Big Three" financial literacy assessment [16], failed to serve as a protective factor against problematic speculative behavior. Conversely, this advanced financial acumen may have functioned as a risk factor, predisposing the individual to engage in *gamblified* investment products [5]. These financial instruments, characterized by high volatility and the potential for rapid capital appreciation or depreciation [5], may have appealed to the patient's overconfidence in his ability to navigate complex financial markets. This phenomenon underscores the nuanced relationship between financial knowledge and risk-taking behavior, suggesting that cognitive biases and psychological factors may override rational decision-making processes even in financially literate individuals. Interestingly, high-risk financial instruments can elicit neurological responses akin to those observed in traditional gambling activities, potentially fostering addictive behaviors [3-7]. The patient's successful transition to more conservative investment strategies and the improvement in his psychometric scores following CBT underscore the potential efficacy of this intervention in mitigating the detrimental effects of *gamblified *investing; however, it is crucial to note that, although the posttreatment NODS score showed improvement, it still established an at-risk status, thereby necessitating ongoing monitoring and support.

**Figure 2:**
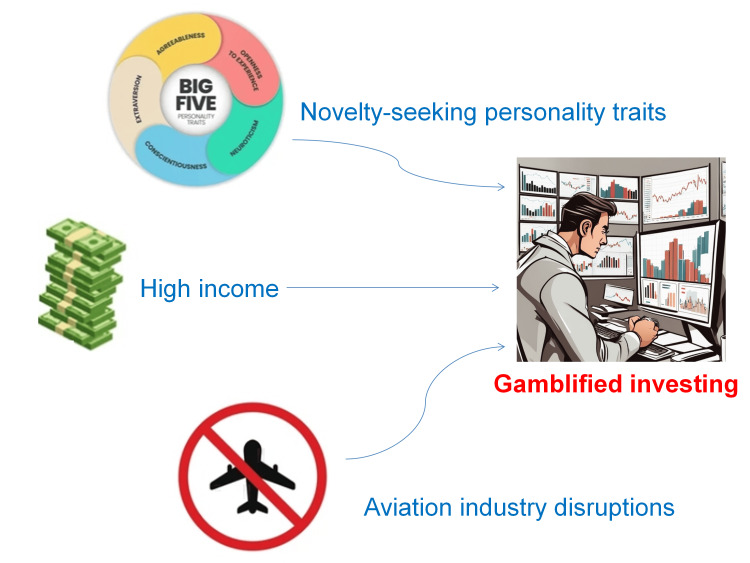
Potential factors contributing to problematic financial behaviors among airline pilots. This schematic diagram delineates the key factors potentially influencing airline pilots' engagement in problematic financial behaviors. Inherent novelty-seeking personality traits may predispose certain individuals to financial risk-taking tendencies. Furthermore, the comparatively substantial remuneration afforded to commercial pilots may facilitate greater access to high-risk financial ventures. Finally, industry disruptions, such as those experienced during the COVID-19 pandemic and anticipated future challenges related to climate change, may result in increased discretionary time. This surplus of unstructured hours could potentially exacerbate risk-taking behaviors, culminating in a propensity toward *gamblified* investment practices. Image credit: Piercarlo Minoretti.

Several factors may predispose airline pilots to problematic financial behaviors. First, the presence of novelty-seeking personality traits among pilots, such as high extraversion scores, particularly in facets like assertiveness, activity, and excitement-seeking [9], may contribute to increased risk-taking in financial decisions. Second, the relatively high salaries of commercial pilots may provide more opportunities for engaging in high-risk financial activities. Third, aviation industry disruptions, like those recently experienced due to the COVID-19 pandemic [17] and potentially in the future due to climate change [18], may lead to a peculiar set of circumstances, including increased free time, which may exacerbate risk-taking behaviors (Figure 2).

Given the safety-critical nature of piloting, addressing potentially addictive financial behaviors is crucial not only for individual well-being but also for public safety. This case highlights the potential benefits of incorporating validated tools like the NODS into regular occupational health evaluations. Furthermore, it emphasizes the necessity for a comprehensive, multidisciplinary strategy to address problematic gambling in safety-critical professions like aviation. Experts from psychology, medicine, sociology, economics, aviation safety, and education should collaborate to understand the complex factors contributing to gambling addiction. By combining their expertise, they can develop effective interventions, policies, and support systems to mitigate these risks. This approach should include enhanced screening protocols for both traditional gambling and high-risk financial activities, tailored psychotherapeutic interventions compatible with pilots' schedules, educational programs on the risks of *gamblified* investment products, especially for those with high financial literacy, and regular monitoring and follow-up to prevent relapse.

## Conclusions

This case report elucidates the potential for high-risk financial behaviors to manifest as a form of behavioral addiction, particularly within the context of occupational stress and disruption. It underscores the imperative for enhanced awareness, targeted screening protocols, and tailored interventions within occupational health settings, with a specific emphasis on safety-critical professions such as commercial aviation. Future research endeavors should prioritize the development of more comprehensive screening instruments capable of early identification of problematic financial behaviors. Furthermore, investigations into the long-term efficacy of therapeutic modalities, such as CBT, in managing these behaviors are warranted. Additionally, empirical studies examining the prevalence of high-risk financial behaviors and pathological gambling among aviators, as well as their potential ramifications on flight safety, would contribute significantly to the existing body of knowledge in this domain.
